# Impact of Radiotherapy on Daily Function Among Older Adults Living with Advanced Cancer

**DOI:** 10.1093/geroni/igaa057.3277

**Published:** 2020-12-16

**Authors:** Anthony Nehlsen, Parul Agarwal, Madhu Mazumdar, Kavita Dharmarajan

**Affiliations:** Mount Sinai School of Medicine, New York, New York, United States

## Abstract

Radiation therapy (RT) improves quality of life and symptomatic burden for patients with advanced malignancies. However, RT can also confer toxicity and little is known about the contribution of geriatric conditions to RT-related outcomes in older adults. This study aims to examine changes in daily function among RT patients at 1 and 6 months following RT. We reviewed charts of 137 patients who underwent RT with intent to improve daily functioning. ADL and IADL scores ranging from 0-6 and 0-8, respectively were collected at baseline, 1-, and 6-months post-RT. Latent class analysis of baseline ADL and IADL was conducted to categorize patients into two classes (high and low deficit). Latent transition analysis was used to examine transitions at each time point. One-hundred seventy courses of RT were identified; 99 were fully evaluable. Median age was 66 years. For ADL and IADL, at baseline 28.9% and 28.3% were classified as high deficit and 71.1%, 71.3% as low deficit respectively; 2% and 7% of low deficit patients had the potential to move to high deficit group at 1 month; and 20% and 13% had the potential to have the same movement from 1 to 6 months. All patients classified as high deficit for both measures at 1 month remained so at 6 months. ADL and IADL functioning may be useful in describing changes in daily function after and identifying groups of patients who may benefit from additional supportive geriatric and/or palliative care interventions.

